# Pharmacotherapy of Posttraumatic Cognitive Impairments

**DOI:** 10.1155/2006/460592

**Published:** 2006-05-11

**Authors:** David B. Arciniegas, Jonathan M. Silver

**Affiliations:** ^1^Brain Injury Rehabilitation UnitHealthONE Spalding Rehabilitation HospitalAuroraCOUSA; ^2^Neuropsychiatry ServiceDepartment of PsychiatryUniversity of Colorado School of MedicineDenverCOUSA; ^3^Behavioral Neurology SectionDepartment of NeurologyUniversity of Colorado School of MedicineDenverCOUSA; ^4^New York University School of MedicineNew YorkNYUSA

**Keywords:** Traumatic brain injury, glutamate, dopamine, acetylcholine, stimulants, cholinesterase inhibitors

## Abstract

Pharmacotherapy may contribute to the rehabilitation of persons with posttraumatic cognitive impairments. This article reviews first the neurobiological consequences of traumatic brain injury with a particular emphasis on acute and long-term posttraumatic neurochemical disturbances. Studies of pharmacotherapies for posttraumatic cognitive impairments are reviewed next, and are organized according to medication class and the neurotransmitter system they affect most. Based on the evidence provided by that review, augmentation of posttraumatic cerebral catecholaminergic and cholinergic function are suggested as potentially useful neurochemical targets for pharmacologic intervention in this population. More specifically, it is suggested that persons with posttraumatic impairments in arousal, speed of processing, and possibly attention may benefit most from treatment with an agent that augments cerebral catecholaminergic function, and that persons whose predominant posttraumatic impairment is in the domain of memory may benefit most from treatment with cholinesterase inhibitors. Practical considerations regarding the use of pharmacotherapies for posttraumatic cognitive impairments are offered, and the need for additional research in this area is highlighted.

## 1. Introduction

Cognitive impairments are among the most common consequences of traumatic brain injury (TBI) at all levels of severity. Although persons with TBI may experience impairments in any cognitive domain, posttraumatic cognitive impairments most often include disturbances of arousal, diminished speed of information processing, attentional and/or memory impairments, language and social communication disturbances, and executive dysfunction [4,100,155, 156,175]. Such impairments may arise as a function of direct injury to cortical, subcortical, or brainstem elements of the distributed cerebral networks that support normal cognitive function [91,127,188], the axonal connections within or between these networks [91,106, 127,128,188], and/or the afferent neurochemical projections that participate in the modulation of function within these networks [41,106,112]. Although neurobiological consequences of TBI remain incompletely understood, clinicians working with persons with TBI will be well advised to stay apprised of this rapidly growing body of information in order to make informed decisions regarding the use of pharmacologic agents in this population [4,115,127].

Toward that end, this article provides first a brief review of neurobiology of posttraumatic cognitive impairments, and then a review of present pharmacotherapies for such impairments. This review was predicated on initial searches of the medical literature in PubMed and Medline using the terms “traumatic brain injury,”

ISSN 0953-4180/06/$17.00 © 2006 – IOS Press and the authors. All rights reserved

"brain injury," "brain injuries," "closed head injury," "head injury(ies)," "craniocerebral trauma," and "concussion." The review of the neurobiology of posttraumatic cognitive impairments included information derived from both human and animal studies, and was anchored to terms relevant to the neuropathological, neuroanatomic, neurochemical, and cognitive consequences of TBI. The review of pharmacotherapies was limited to studies undertaken among adults with traumatic brain injuries only, and was anchored to the classes of pharmacologic agents into which this article was organized.

## 2. The neurobiological bases of cognitive impairments following TBI

TBI produces a complex cascade of potentially injurious processes including focal contusions, diffuse axonal injury, cytotoxic damage, and neurotransmitter excitotoxicity. Focal contusions are widely acknowledged to be common consequences of severe TBI, but are produced more commonly by mild TBI than is often acknowledged by clinicians unfamiliar with this population [17,70,71,109,143,146,159]. Focal traumatic lesions tend to produce severe and persistent impairments of the cognitive functions served by the brain areas in which they are located; for example, executive impairments following severe bifrontal contusion, impaired anterograde memory following bilateral entorhinal-hippocampal injury, and so on. The development of trauma-related neuroimaging abnormalities among persons with mild TBI (so-called "complicated" mild TBI) appears to affect posttraumatic cognitive outcome in a similar fashion: individuals with such findings after mild TBI experience deficits similar to those among persons with GCS-defined moderate TBI [168, 181].

Cytotoxic processes such as calcium and magnesium dysregulation [104,169], free radical formation [12, 47], excitatory amino acid/neurotransmitter excitotoxicity [18,82,84,116,172,190], and diffuse axonal injury due to straining and shearing biomechanical forces [104,127–129] are common among persons with penetrating and non-penetrating/non-contusional injuries. Among these processes, traumatically-induced axonal injuries are the most studied with respect to their cognitive consequences.

Although axonal injury is traditionally described as "diffuse," the effects of high-speed, long-duration deceleration injuries (as experienced in motor vehicle-related and sports-related TBI) are most evident in axonal projections within and from the brainstem, the parasagittal white matter of the cerebrum, the corpus callosum, and at the gray-white junctions of the cerebral cortex [106]. The clinical consequences of traumatically-induced axonal injuries typically include cognitive slowing, problems with frontally-mediated cognitive functions such as attention, working memory, recall of recently learned declarative information, language (i.e. word-finding problems), and executive function [10,53,59,125,174].

In addition to the mechanical and cytotoxic contributors to TBI, human and animal studies suggest that the application of stretching and straining forces to the brain produces acute and potentially neurotoxic excesses of cerebral neurotransmitter levels. Such excesses include acute elevations of glutamate [82,84, 172,189,190], dopamine [97,183], norepinephrine [69, 97,184], serotonin [97,126], and acetylcholine [72,78, 135,139]. Since neurotransmitter systems are the targets of pharmacotherapies for posttraumatic cognitive impairments, the effects of TBI on each of the above major neurotransmitter systems are considered here in additional detail.

### 2.1. Glutamate

Glutamate is an amino acid that serves as the primary excitatory neurotransmitter in the central nervous system. When glutamate is released in a controlled fashion, it facilitates neuronal activation via its actions at N-methyl-D-aspartate (NMDA), kainite, and a-amino-3-hydroxy-5-methyl-4-isooxazolepropionate (AMPA) receptors. When glutamate activates an NMDA receptor, it opens a channel in the cell membrane that permits influx of calcium and facilitates both neuronal depolarization and a cascade of secondary processes that result in signal transduction. When released in an uncontrolled (excessive) fashion, calcium influx initiates oxidative processes and activation of proteolytic enzymes that injure or destroy the neuron and/or its axonal terminus. Even where glutamate excesses are not directly excitotoxic, they tend to "drive" glucose utilization, which may exceed the capacity of the brain for oxidative metabolism and produce toxic accumulations of lactate [3].

Traumatically-induced glutamate excitotoxicity is in all likelihood an important contributor to injury of areas critical to neuropsychiatric function [82,84,172, 189,190], although consensus on this matter is lacking [116]. Cerebral glutamate levels are elevated quickly (probably within minutes) following TBI and remain elevated throughout the first week following TBI in humans [11,160,189,190]. The degree of excitatory amino acid neurotransmitter elevations after TBI are associated with the severity of injury and inversely associated with post-injury survival [64,75,82]. Although glutamate levels trend towards normalization in the weeks following TBI [116], the rate at which such normalization occurs may be adversely affected by concurrent hypoxia [99]. Animal models suggest that antagonism of glutamate receptors may attenuate the severity of neuronal injury following trauma [20, 84]. Glutamate-mediated excitotoxicity also may be attenuated by therapeutically induced, but not spontaneous, cerebral hypothermia in the acute post-injury period [152]. Unfortunately, there are at present no proven interventions that effectively limit the neurotoxic effects of acute posttraumatic excesses of glutamate among humans [76].

Glutamate-mediated neuronal damage appears to be particularly severe in cortical areas [192]. In those cortical areas in which glutamate is the both the dominant and key neurotransmitter (i.e. hippocampal and frontal cortices), glutamate-mediated excitotoxicity would be expected to contribute to posttraumatic impairments in the cognitive functions served by these areas (i.e. memory and executive functions) [84,134]. However, the relationship between long-term posttraumatic glutamate disturbances and cognitive impairments is not well established. Accordingly, the relevance of agents that modify glutamate signaling to the treatment of posttraumatic cognitive impairments remains uncertain.

### 2.2. Catecholamines

The major cerebral catecholamines dopamine and norepinephrine, serve as key modulators of information processing circuits in the brain [66]. Each of these neurotransmitters acts on multiple receptor types [58, 77]. Their actions at those receptors appear to alter the signal-to-noise ratio in cerebral information processing circuits [66,117,157]. In the setting of optimal catecholamine levels, the processing of contextually relevant cognitive, emotional, or behavioral information (signal) is enhanced and processing of background information (noise) is inhibited. Because dopamine and norepinephrine exhibit an inverted U-shaped dose-response curve with respect to their effects on cortical information processing [105], either deficient or excessive dopamine may interfere with this process.

The basal and anterior frontal pathways followed by ascending catecholaminergic projections places them in anatomical areas that are especially vulnerable to strain and shear forces that occur during TBI and CSF sampling of catecholamine metabolites suggest that TBI produces acute cerebral elevations of these neurotransmitters [97]. Acute catecholaminergic excesses are predictive of poor recovery from TBI [41,69,184]. Whether such excesses are themselves neurotoxic or are instead simply a proxy for injury severity is not clear.

Although traumatically-induced disruptions of ascending catecholaminergic projections would be expected to interfere with cognitive function [111], the role of catecholaminergic dysfunction in the development of persistent posttraumatic cognitive impairments is not established definitively. Both hyperdopaminergic [161] and hypodopaminergic [41,132,178,179, 179,180] function are hypothesized to interfere with cognitive function following TBI. Preliminary findings from studies pairing pharmacologic probes (dopaminergic agonists and alpha-2 adrenergic agonists) with functional magnetic resonance imaging underway in the laboratory of McAllister and colleagues [101] suggest that posttraumatic working memory impairments may be more specifically attributable to dysfunction of cerebral noradrenergic systems. Additionally, genetically determined individual differences in the metabolism of these neurotransmitters by catechol-O-methyltransferase (COMT) [86] may contribute to posttraumatic cognitive impairments [92]. While the contributions of catecholamine dysfunction and individual differences in catecholamine metabolism to such impairments require further investigation, findings from experimental injury studies, investigations of posttraumatic cerebral catecholamine disturbances, and pharmacologic studies employing catecholamine augmentation strategies suggest that these neurotransmitters may be reasonable targets for pharmacotherapies in this population.

### 2.3. Serotonin

Serotonin serves as a modulatory neurotransmitter in the central nervous system. At least 14 serotonin receptor subtypes have been characterized [66,118], each differing with respect to its effects on cerebral function. Serotonergic projections from the midbrain and pontine raphe nuclei to frontal cortical areas are susceptible on anatomic grounds to the same forces that injure catecholaminergic projections. Additionally, serotonergic projections may be damaged biomechanically or by secondary neurotoxic processes (i.e. excitotoxic injury and/or lipid peroxidation [80]). Serotonergic excesses have been observed following TBI in both experimental [22,45] and human [126,170] studies. The principal effect of traumatically-induced elevations of cerebral serotonin appears to be to decreased cerebral glucose utilization [121,167]. As a result of decreasing cortical metabolism, traumatically-induced elevations of serotonin may in part contribute to posttraumatic cognitive impairments during the acute injury period. Consistent with this hypothesis, augmentation of serotonergic function in the acute injury period appears to confer little cognitive benefit [107,182].

It is possible that TBI may produce long-term serotonergic deficits. Persistent posttraumatic serotonergic deficits are likely to be relevant to the development of posttraumatic mood and anxiety disorders, which may themselves contribute to posttraumatic cognitive impairments. Treatment of posttraumatic depression with serotonin reuptake inhibitors may facilitate improvement in depression related cognitive impairments [49, 74] but such effects are not universally associated with these treatments [88]. However, the direct relevance of posttraumatic serotonergic deficits – if they do occur – on posttraumatic cognitive function is uncertain. In fact, the pro-cognitive effects of medications that augment serotonergic function may reflect the effects of these agents on other neurotransmitter systems [142].

Even if TBI does not produce persistent damage to serotonergic systems, it is possible that receptor-specific modulation of serotonergic function (i.e. antagonism of $5HT_{1A}$, $5HT_{1B}$, and $5HT_3$ receptors, or agonism of $5HT_{2A}$, $5HT_{2C}$, and $5HT_4$ receptors) may improve posttraumatic cognitive impairments [19]. Consistent with that suggestion, Kline et al. (2001) [83] demonstrated improvement in spatial memory in experimentally injured rats treated with the $5HT_{1A}$ receptor antagonist repinotan. If similar findings are observed in humans with posttraumatic cognitive impairments, agents targeting specific serotonin receptors may develop as a useful treatment strategy in this population.

### *2.4. Acetylcholine*

Acetylcholine is a ubiquitous cerebral neurotransmitter that acts as a modulator of excitatory and inhibitory neurotransmission [66,94]. Cholinergic projections to the brain have their origins in several discrete basal forebrain and brainstem nuclear groups, the projections from which remain segregated as they ascend to their cortical target areas [145]. Receptors for acetylcholine are classified into muscarinic and nicotinic types, each of which has multiple subtypes that display different intrinsic and regional functional properties [51,138,149]. Although acetylcholine plays an important role in many cognitive functions, it appears to play a key role in the development of normal arousal and attention [15,105] and of memory [1,15,60].

TBI produces acute elevations and long-term deficits in cortical cholinergic function [29,33,36–40,139]; these studies also support a robust relationship between chronic posttraumatic cholinergic deficits and memory impairments. In comparison to catecholaminergic and serotonergic projections, cholinergic projections also appear to be particularly susceptible to damage by traumatic mechanical forces [141]. The destructive effects of acute elevations of cortical acetylcholine (Ach) may be reduced in animal models by pre-administration of the muscarinic antagonist, scopolamine [95,139]. In the absence of adequate pre-injury neuroprotection from cholinergic excitotoxicity, concussed animals develop chronic reductions in cholinergic function and remain susceptible to worsening of cognitive function during treatment with anticholinergic agents [38,40].

TBI in humans produces a similar pattern of posttraumatic cholinergic dysfunction, with acute elevations of cerebral acetylcholine [65] followed by chronic damage to cerebral cholinergic nuclei [140], projections [34,112,113], and cholinergically-dependent information processing circuits [5–8]. Collectively, these findings suggest that cholinergic function is chronically deficient among many persons with TBI and that cholinergic deficit may be a significant contributor to posttraumatic cognitive impairments, and particularly memory impairments. As such, posttraumatic cholinergic deficits may be a useful target for the pharmacotherapy of posttraumatic cognitive impairments [5,8, 32,177].

## 3. Summary of the neurobiological bases of posttraumatic cognitive impairments

TBI produces a complex set of processes that are injurious to brain areas serving cognition. Focal contusions appear to produce the most severe and persistent cognitive impairments, the types of which are relatively easily understood on the basis of our present understanding of the neuroanatomy of cognition. Axonal injuries, in combination with the cytotoxic processes associated with such injuries, are in all likelihood the most common anatomic contributor to posttraumatic cognitive impairments, and have their greatest effects on the speed and efficiency of information processing. Stretching and straining of axons also produces acute elevations of most cerebral neurotransmitter levels. The present evidence suggests that traumatically-induced excesses of glutamate and acetylcholine, and perhaps the catecholamines, are neurotoxic. The destructive effects of such neurotransmitter excesses appear to be greatest in the areas in which these neurotransmitters are co-localized, and particularly in the basal forebrain, hippocampus, striatum, and frontal cortices. Neurotransmitter-induced excitotoxic injuries would be expected to produce impairments in the cognitive functions supported by these areas, including arousal, attention, memory, and executive function. As the acute neurotransmitter "storm" abates, long-term deficits develop in cerebral cholinergic systems and possibly also in catecholaminergic systems. Deficits in these systems would be expected to maintain impairments in the cognitive functions they support: arousal, speed of processing, attention, memory, and executive function. The pharmacotherapy of TBI will be considered in the context of these findings.

## 4. Pharmacotherapy of posttraumatic cognitive impairments

### *4.1. General considerations*

A thorough history and examination is a pre-requisite to the prescription of any pharmacologic treatment for posttraumatic cognitive impairments. The potential contributions of other common posttraumatic problems such as headache, cervical or other somatic sources of pain, sleep disturbance, fatigue, mood, and anxiety symptoms should be addressed. Treatment of these conditions should be considered prior to prescribing treatments for posttraumatic cognitive impairments more specifically.

When subsequently considering use of pharmacotherapies for posttraumatic cognitive impairments, two issues require additional attention. First, the presenting cognitive complaints must be carefully assessed, defined, and operationalized, preferably though the use of objective measures of cognition. Although "bedside" tests of cognition may be useful for this purpose, posttraumatic cognitive impairments are better assessed through formal neuropsychological testing. Second, the use and effectiveness of all ongoing treatments must be frequently reevaluated, including both pharmacologic and nonpharmacologic therapies (whether prescribed or self-administered). Anticonvulsants such as phenytoin and carbamazepine may exacerbate cognitive impairments among persons with TBI [35,151]; absent the development of posttraumatic seizures, severe mood disturbances, or intractable aggression requiring their use, prescription of anticonvulsant agents should be avoided whenever possible. Typical antipsychotic medications (e.g. haloperidol, fluphenazine, thioridazine, chlorpromazine) may exacerbate cognitive impairments among persons with TBI [158] and may prolong the period of posttraumatic amnesia [132]. Benzodiazepines are known to impair memory and other aspects of cognition [18], and appear to do so among persons with TBI [14]. Additionally, these classes of pharmacologic agents may impede neuronal recovery after TBI [63]. Where possible, these agents should be avoided, eliminated, or at least reduced prior to initiating treatment with an agent specifically targeting posttraumatic cognitive impairments.

If after reducing or eliminating medications that may be contributing to posttraumatic cognitive impairments there remain cognitive problems that require treatment, then specific therapies for such impairments are appropriate to offer. Nonpharmacologic therapies should be included as a part of any treatment plan for posttraumatic cognitive impairments; clinicians may wish to review the recommendations offered by Cicerone et al. (2000) [30] regarding the use of cognitive rehabilitation among persons with TBI. When these interventions fail to afford needed improvement in posttraumatic cognitive performance, or when a combined pharmacological-cognitive rehabilitation approach is preferred, then medications may be of use in the treatment of posttraumatic cognitive impairments.

Where possible, clinicians should predicate the treatments they offer on the published literature specific to TBI. The majority of the treatment literature regarding posttraumatic cognitive impairments consists of open-label case series, single case reports, and a few single-site, randomized, double-blind, placebo-controlled studies [4,102]. Absent published studies with which to guide treatment selection, development of an *a priori* hypothesized therapeutic rationale relevant to the neurochemical bases of posttraumatic cognitive impairments is recommended. Unfortunately, simple, inexpensive, and widely available markers of *in vivo* neurotransmitter function are presently lacking, leaving clinicians the task of selecting pharmacotherapies on the basis of the hypothesized link between neurotransmitter dysfunction and the most prominent cognitive impairments experienced an individual patient. For example, posttraumatic impairments in arousal and speed of processing would be expected to respond best to catecholaminergic augmentation. Impairments in attention and executive function might respond to agents that improve catecholaminergic or cholinergic function, or both. Posttraumatic memory impairments would be expected to respond best to cholinergic augmentation strategies. While necessarily speculative, the evidence described in the following sections of this manuscript supports reasonably well this approach to the pharmacotherapy of posttraumatic cognitive impairments.

Given this background, pharmacotherapies for posttraumatic cognitive impairments are presented here according to medication class and the neurotransmitter system(s) that they augment most strongly. Guided by the published literature and our own clinical experience, we offer preliminary recommendations regarding the pharmacotherapy of posttraumatic cognitive impairments. It is important for clinicians to remain mindful of the fact that no medication has yet received approval from the United States Food and Drug Administration (FDA) for the treatment of any neuropsychiatric consequence of TBI, including cognitive impairments. Consequently, clinicians must regard all of the treatments described in this article as "off-label." We encourage clinicians to consider the application of these agents to the treatment of an individual patient a matter of empiric trial.

## 5. Dopaminergic augmentation strategies

### *5.1. Bromocriptine*

Bromocriptine appears to act directly on postsynaptic dopamine type 2 (D2) receptors. At low doses, bromocriptine acts as a pre-synaptic D2 agonist, and thereby reduces dopaminergic release and function in dopaminergically mediated systems. Its net effect at mid-range doses appears to augment the function of cerebral dopaminergic systems [13]. Passler et al. (2001) [122] observed bromocriptine treatment-related improvements in arousal (i.e. transition from persistent vegetative state to minimally conscious state) among 5 subjects with TBI. Eames (1989) [43] and Powell et al. (1996) [130] suggest that bromocriptine may be useful in treating "cognitive initiation" problems (i.e. apathy) in the late post-injury period. McDowell et al. (1998) [103], using a counterbalanced, double-blind, placebo-controlled, crossover design in twenty-four subjects with TBI, observed improved performance on executive function during treatment with bromocriptine but no improvement on other posttraumatic cognitive impairments. Although these studies are suggestive of a possible benefit of bromocriptine on posttraumatic impairments of arousal and frontally mediated functions (e.g. motivation, executive function), large-scales studies evaluating the safety and efficacy of bromocriptine on posttraumatic cognitive impairments are not available.

Treatment with bromocriptine generally begins with 2.5 mg/day and is gradually titrated to the highest dose tolerated. Dizziness, drowsiness, faintness, syncope, nausea, vomiting, abdominal cramps, constipation, and diarrhea are relatively common side effects, but are generally of mild severity. Uncontrolled hypertension and hypersensitivity to ergot alkaloids are strict contraindications to the use of bromocriptine. Use of this agent is contraindicated among women breastfeeding their infants, but use during pregnancy does not appear to be associated with significant increased adverse events among pregnant patients or on fetal development. Bromocriptine may possess some anticonvulsant properties [137], offering some reassurance of its safety for use among cognitively impaired TBI survivors with this problem.

### *5.2. Amantadine and memantine*

Amantadine and memantine are moderate-affinity uncompetitive NMDA receptor antagonists. Although these agents are of theoretical interest with respect to both the prevention of traumatically-induced glutamate excitotoxicity [133] and the remediation of posttraumatic cognitive impairments, the relevance of their effects on glutamatergic signaling in this context is uncertain. Since both of these agents appear to increase dopamine release, decrease presynaptic dopamine reuptake, stimulate dopamine receptors, and/or enhance postsynaptic dopamine receptor sensitivity [31,120, 123,124,131,150,153], their therapeutic effects are in all likelihood best attributed to these properties.

There are, at present, no published studies regarding the use of memantine for the treatment of posttraumatic cognitive impairments. Several studies describe benefits afforded by amantadine on posttraumatic cognitive impairments and frontally-mediated behavioral disturbances [26,67,85,114,171]. In the largest study of this agent for posttraumatic cognitive impairments, Meythaler et al. (2002) [108] performed a

12-week, double-blind, placebo-controlled, crossover design study of amantadine 200 mg daily in thirty-five subjects with severe TBI during the acute post-injury period. They observed treatment-related improvements in Mini-Mental Status Examination scores, Disability Rating Scale scores, Glasgow Outcome Scale scores, and in the cognitive domain score of the Functional Independence Measure (FIM). They suggest that amantadine may improve acute recovery following TBI regardless of the precise timing of treatment during the subacute post-injury period.

Amantadine is generally started at a dose of 50 mg twice daily, and is usually increased every week by 100 mg/day to either symptomatic improvement or medication intolerance. Amantadine 100 mg twice daily is often sufficient to impart improvement in these symptoms without undue side effects; in any case, the maximum dosage of amantadine should not exceed 400 mg daily. Common side effects include headache, nausea, diarrhea, constipation, anorexia, dizziness, lightheadedness, orthostatic hypotension. Anxiety, irritability, depression, and hallucinations may also develop during treatment with this agent, but are relatively uncommon. At higher doses, psychosis and confusion may occur. Abrupt withdrawal of this agent has been associated (rarely) with neuroleptic malignant syndrome. Additionally, co-administration of triamterene/hydrochlorothiazide may decrease renal excretion of amantadine, resulting in medication intolerance at doses that would ordinarily be regarded as within the usual therapeutic range. Amantadine also potentiates the effects of agents with anticholinergic properties. Adverse reactions to amantadine appear to occur more often in elderly patients than in younger patients. It has been reported that amantadine may lower seizure threshold [67], although studies of amantadine in persons with refractory epilepsy offer only modest support for this suggestion [42,147]. Nonetheless, clinicians are advised to be vigilant for the development or worsening of seizures when amantadine is used among persons with TBI.

### *5.3. Carbidopa/L-Dopa*

L-dopa (levodopa) is a dopamine precursor that is usually co-administered with carbidopa, which serves to decrease the extent of its metabolism in the periphery. Although this medication is uncommonly used in clinical practice for the treatment of posttraumatic cognitive impairments, Lal et al. (1988) [87] observed improvements in alertness and concentration, decreased fatigue, hypomania, and sialorrhea, as well as improved memory, mobility, posture, and speech among 12 persons with brain injury (including several patients with hypoxic-ischemic brain injuries) treated with carbidopa/L-dopa 10/100 to 25/250 four times daily. However, additional and larger studies evaluating the efficacy and safety of this agent should be conducted before recommending its routine use in this population.

If carbidopa/L-dopa is used, treatment should begin with low doses (25/100 twice daily) and gradually increase to doses of 25/250 four times daily. Common side effects are predominantly related to its central dopaminergic effects and may include dykinesias, anxiety, hallucinations (especially visual), paranoia or overt psychosis. Nausea may be a treatment-limiting side effect in some patients and is more common during treatment with preparations of relatively lower doses of carbidopa (i.e. 10/100 vs. 25/100). Less frequent side effects include palpitations, orthostatic hypotension, anorexia, vomiting, and dizziness. Rare but serious adverse effects include gastrointestinal hemorrhaging, duodenal ulcer, hypertension, phlebitis, leukopenia, agranulcytosis, thrombocytopenia, and hemolytic or nonhemolytic anemia. Carbidopa/L-dopa does not appear to reduce seizure threshold clinically, but there is insufficient data with which to assess the risk of seizures during treatment with this agent among persons with TBI.

### *5.4. Mixed catecholaminergic augmentation strategies* (*Psychostimulants*)

Methylphenidate and dextroamphetamine increase the release of both dopamine and norepinephrine and, at higher doses, block the reuptake of these monoamines. These agents also modestly inhibit monoamine oxidase, which in combination with their other effects increases the effectiveness of monoaminergic neurotransmission. Although increasing cerebral catecholaminergic activity would be expected to improve many domains of cognitive function, in practice the effects of catecholaminergically active agents appear to be most robust in the domains of arousal, speed of processing, and attention.

#### *5.4.a. Methylphenidate*

Early reports suggested that methylphenidate may improve posttraumatic impairments in arousal, cognitive and motor speed, attention, memory, mood, and some aspects of behavior, as well as the rate (although not ultimate level) of functional recovery [48, 68,79,125,154,185]. Whyte et al. (1997) [180], using a randomized, double-blind, placebo-controlled, repeated crossover design in nineteen subjects with remote nonpenetrating TBI recruited from either inpatient or outpatient settings, observed a significant effect of methylphenidate on arousal and speed of processing but no improvements on most aspects of vigilance (sustained attention), distraction, or motor speed. In a detailed critical review of the literature, Whyte et al. (2002) [179] concluded that the principal benefits of methylphenidate are on posttraumatic impairments of processing speed and to a lesser extent on subjective ratings of behavior and mood. Whyte et al. (2004) [178] subsequently evaluated the effects of methylphenidate on a variety of aspects of attention among 34 adults with moderate-to-severe TBI and attention complaints in the postacute injury period. Using a 6-week, double-blind, placebo-controlled, repeated crossover study of methylphenidate 0.3 mg/kg/dose twice daily, they observed a treatment effect on 5 of 13 attentional factors in a pilot sample of ten subjects. In only three of these factors (speed of information processing, attentiveness during individual work tasks, and caregiver ratings of attention) were statistically significant treatment effects observed in a replication sample of twenty-four subjects. In a subsequent analysis, only reaction time before errors demonstrated a significant treatment effect in the replication sample. They observed no treatment-related improvements in divided attention, sustained attention, or susceptibility to distraction. Conversely, no negative effects of treatment on cognition were observed. Although findings from this study appear to contradict earlier study findings and the common clinical use of methylphenidate for impairments of vigilance and distractibility, these studies are the most carefully constructed and systematically analyzed of its kind in the TBI literature, and seem to suggest that the primary benefit of methylphenidate is on posttraumatic impairments of processing speed. Although it stands to reason that improvements in processing speed afforded by methylphenidate might secondarily improve attention, memory, and other "higher" cognitive functions, the studies performed to date offer little support for this suggestion.

*5.4.b. Dextroamphetamine*

Dextroamphetamine is chemically similar to methylphenidate and is sometimes used to treat posttraumatic impairments of arousal, speed of processing, attention and memory. However, the evidence to support its use for these purposes is sparse, at best [14,48,73]. Although encouraging of a role for dextroamphetamine for the treatment of posttraumatic cognitive impairments, additional studies are needed to better define the usefulness of this agent for this purpose and to compare its therapeutic equivalence and/or superiority (or lack thereof) to methylphenidate.

*5.4.c. Use of psychostimulants in clinical practice*

To the extent that an individual's posttraumatic cognitive impairments are related to catecholaminergic dysfunction, methylphenidate and dextroamphetamine may afford some degree of improvement in speed of processing, and possibly arousal and attention. In clinical practice, careful assessment of arousal, speed of processing, and attention should be undertaken before and during treatment with these agents. In the absence of objective improvement during treatment with these agents, reports of subjective improvement in cognition or daily function during treatment with these agents are frequently used as a measure of treatment response. However, subjective reports of improvement in the absence of improvement on objective measures of cognitive should prompt re-evaluation of the etiology of those impairments. Since psychostimulants may improve mood, increase motivation, or lessen fatigue, discrepancy between subjective and objective measures of improvements may suggest that the primary problem lies in posttraumatic depression, apathy, fatigue, or some combination of these non-cognitive problems.

Stimulants generally take effect quickly (within 0.5–1 hour following administration) and also lose effect after only a few hours. Therefore, the first issue in the administration of these agents is determining optimal dose and dosing frequency. Initial dosing with either agent generally begins at 5 mg twice daily and is gradually increased in increments of 5 mg twice daily until either beneficial effect or medication intolerance is achieved. Most studies suggest that optimal doses of either methylphenidate or dextroamphetamine are in the range of 20–40 mg twice daily (i.e. 0.15–0.3 mg/kg/twice daily). Individuals requiring relatively high and frequent doses of methylphenidate or dextroamphetamine may benefit from use of longer-acting preparations of these medications.

Mild increases in heart rate and/or blood pressure may occur during treatment with psychostimulants, although these tend to occur relatively infrequently in patients without other cardiac or vascular problems and are only rarely of sufficient magnitude to merit discontinuation of these agents [2,21]. Use of these agents during pregnancy is discouraged, and their use is contraindicated among patients receiving monoamine oxidase inhibitors (MAOIs) and women who are breastfeeding. Clinicians are advised to avoid use of these agents among persons with comorbid Tourette's syndrome and other tics, glaucoma, untreated hypertension or cardiovascular problems, and symptomatic hyperthyroidism. These agents may potentiate the effects of Phenobarbital and phenytoin by delaying their gastrointestinal absorption; increasing the noradrenergic effects of tricyclic antidepressants; increas the dopaminergic effects of antiparkinsonian agents; and potentiate the analgesic properties of meperidine. Because depressed mood and increased fatigue may develop following abrupt discontinuation of psychostimulants, these medications should be discontinued gradually among patients receiving them chronically. Serious adverse reactions to these medications are most often related to increases in cerebral dopamine, and to a lesser extent cerebral norepinephrine activity, and include paranoia, dysphoria, anxiety, agitation, and irritability. However, these adverse effects are in practice very uncommon at doses typically used to treat posttraumatic cognitive impairments. Although lowering seizure threshold is often cited as a risk associated with the use of these agents, this risk appears to be minimal [186], even among persons with epilepsy [52,93].

## 6. Other stimulant-like agents

### 6.1. Modafinil

Modafinil, a medication approved for the treatment of excessive daytime somnolence in patients with narcolepsy, may have a role in treatment of post-TBI fatigue and cognitive impairment. The exact mechanisms of action of modafinil are not understood fully, but may include activation of hypocretin (orexin) neurons in the lateral hypothalamus [27], indirect dose-dependent reductions in gamma-aminobutyric acid (GABA) release in the cerebral cortex, medial preoptic area, and posterior hypothalamus [54,162], dose-dependent increases in glutamate release in the ventrolateral and the ventromedial thalamus [55], and/or increases in dopamine in the nucleus accumbens [56]. Among persons with attention deficit hyperactivity disorder, modafinil appears to be similarly effective to dextroamphetamine [164]. Elovic (2000) [46] suggest that modafinil may be beneficial for posttraumatic impairments in arousal, and Teitelman (2001) [165] observed improvements in arousal and attention after TBI in an open-label study of 10 persons with TBI treated with this agent in an outpatient setting. Although these findings are encouraging of a role for modafinil in the treatment of posttraumatic cognitive impairments, further studies of this agent for this purpose are needed before any recommendations regarding its use are offered.

### 6.2. Protriptyline

Although the anticholinergic and antihistaminergic effects of tricyclic antidepressants would be expected to worsen posttraumatic cognitive impairments, it has been suggested that protriptyline, a secondary amine tricyclic agent, may possess sufficient stimulant properties to permit its use for anergia and diminished motivation in TBI patients [187]. However, there is no evidence that this agent confers any benefit on cognition beyond that afforded by improved arousal and motivation.

### 6.3. Lamotrigine

Lamotrigine is an anticonvulsant agent that may have activating effects, although the mechanism by which lamotrigine confers such benefits is unclear. Showalter and Kimmel (2000) [148] observed improvements in arousal among 9 of 13 persons with severe TBI (Rancho Los Amigos Scale I-III) during the postacute recovery period, and hypothesized that lamotrigine's ability to block sodium channels and inhibit glutamate release may either prevent excitotoxic injury and/or facilitate recovery from injury. Pachet et al. (2003) [119] observed improvements in arousal in a single-case study of lamotrigine used in the late post-injury period following severe TBI. Additional studies are needed to determine whether there is a role for lamotrigine in the treatment of posttraumatic cognitive impairments.

## 7. Cholinesterase inhibitors

This class of medication includes physostigmine, tacrine, donepezil, rivastigmine, and galantamine. Although these agents differ in their central (i.e. cerebral) selectivity and possible additional mechanisms of action [50,96], all of these agents principally exert their clinical effects via inhibition of synaptic acetylcholinesterase.

### 7.1. Physostigmine

Several reports describe cognitive improvements following administration of physostigmine, both in the acute [16] and postacute [44,61,62] injury period. In a double-blind, placebo-controlled study of physostigmine and/or lecithin in 16 persons with cognitive impairment following moderate-to-severe TBI, Levin et al. (1986) [89] observed improvements in sustained attention during treatment with physostigmine. In a double-blind, placebo-controlled, crossover design study of physostigmine, placebo, and scopolamine among 36 males with posttraumatic memory impairment of at least 3 months, Cardenas et al. (1994) [28] observed improvements on the long-term storage component of the Selective Reminding Test in 44% of subjects during treatment with oral physostigmine but not placebo or scopolamine.

Although physostigmine may be of benefit to cognitively impaired TBI survivors, its lack of central selectivity, unpredictable oral absorption, and need to be administered multiple times daily limit its acceptability as a treatment for posttraumatic cognitive impairments. Given the availability of newer and generally better tolerated cholinesterase inhibitors, we do not recommend routine use of physostigmine among persons with posttraumatic cognitive impairments.

### 7.2. Donepezil, rivastigmine, and galantamine

Donepezil is a cholinesterase inhibitor that exhibits relative central selectivity, and its predominant mechanism of action is inhibition of synaptic acetylcholinesterase [28,136]. Zhang et al. (2004) [191] performed a 24-week, randomized, placebo-controlled, double-blind, crossover trial of donepezil, 10 mg daily, in eighteen subjects with TBI 2–24 months post-injury. They observed significant improvements in attention and memory as a function of treatment, regardless of the timing of the delivery of treatment during the postacute recovery period. The group treated with donepezil prior to placebo also demonstrated cognitive improvements at the end of the placebo phase when compared to baseline, suggesting a possible carry-over effect of donepezil on cognitive performance in this study population. Walker et al. (2004) [173] performed a retrospective, age- and injury-severity matched, mixed between-within subjects analysis of the effects of donepezil, 5 mg daily, on posttraumatic memory impairments among thirty-six subjects with moderate-to-severe TBI admitted to acute rehabilitation within 90 days of injury. Although no differences in cognitive improvement were observed between the donepezil treatment group and the matched control group, subset analyses suggested that donepezil improved the rate of recovery of functional cognition (measured by the cognitive subscale of the FIM).

Several relatively small open-label case series suggest that donepezil 5–10 mg daily may also improve posttraumatic memory impairments in the late post-injury period [81,98,163,166,176]. A randomized, open-label, case series of one hundred eleven outpatient subjects with chronic posttraumatic cognitive impairments observed subjectively-reported improvements in attention and “general function” among 61% of subjects. Response to treatment was reportedly rapid and occurred during treatment with relatively low doses of these agents, and no significant differences in effects or tolerability between these agents were observed. Finally, Morey et al. (2003) [110] performed a prospective double-blind, placebo-controlled, crossover design study of donepezil for chronic posttraumatic memory impairments in seven subjects. Although overall treatment effects were modest, they observed significant improvements in immediate and delayed memory during treatment with donepezil, 10 mg per day, but not during treatment with donepezil, 5 mg per day.

Although mixed with respect to the magnitude of their effects, studies of physostigmine, donepezil, rivastigmine, and galantamine suggest these agents may be of benefit for the treatment of posttraumatic memory impairments, and perhaps also for posttraumatic attentional impairments. Posttraumatic cholinergic deficits are common but not universal [9,34,112,113]. Accordingly, the extent to which these agents will be useful in an individual patient most likely depends on the extent to which cholinergic dysfunction contributes to that individual’s posttraumatic cognitive impairments. Multicenter, double-blind, placebo-controlled trials are needed to define the clinical profile of persons with TBI most likely to respond to treatment with a cholinesterase inhibitor, the relative safety and efficacy of these agents in this population, and optimal dosing strategies.

### 7.3. Cholinesterase inhibitors in clinical practice

Donepezil is prescribed most commonly for posttraumatic cognitive impairments; when used, treatment begins with 5 mg daily. When higher-dose donepezil is used, titration is generally undertaken at intervals of 2–4 weeks. Slower dose titration may limit the development of treatment-emergent side effects, which are gastrointestinal in nature. Rivastigmine and galantamine have shorter half-lives, and require twice daily dosing. Rivastigmine is generally started at 1.5 mg BID and increased in 1.5 mg BID increments every four weeks until maximal benefits are attained or treatment intolerance develops. Galantamine is generally started at 4 mg BID and increased in 4 mg BID increments until maximal benefits are attained or treatment intolerance develops.

All of these agents produce with variable frequency, headache, nausea, diarrhea, vomiting, fatigue, insomnia, muscle cramping, pain, and abnormal dreams. These side effects are frequently a consequence of overly rapid dose escalation, although they will occur in a minority of patients during treatment with standard dosing strategies. When intolerable side effects develop and/or persist during treatment with any of these agents, dose reduction is prudent. Such reductions may reduce adverse effects and permit patients to continue treatment.

Use of these agents should be avoided in women who are pregnant or who are breast feeding their children. Cardiac conduction abnormalities (first-degree A-V block) and symptomatic bradycardia are relative contraindications to the use of these medications. Concurrent administration of agents that inhibit hepatic metabolism via CYP450, 3A4, and 2D6 enzymatic pathways (e.g. ketoconazole and quinidine) may increase blood levels of donepezil. Inducers of hepatic metabolism (phenobarbital, phenytoin, carbamazepine, dexamethasone, rifampin) may decrease therapeutic blood levels. To-date, there are no reports suggesting that the use of these agents in persons with traumatic brain injury is associated with a change (positive or negative) in seizure frequency.

## 8. CDP-choline

Cytidine 5'-diphosphocholine (CDP-choline or citicoline) is an essential intermediate in the biosynthetic pathway of phospholipids incorporated into cell membranes. Orally ingested CDP-choline is metabolized into its two principal components, cytidine and choline. CDP-choline appears to activate the biosynthesis of structural phospholipids in neuronal membranes, increase cerebral metabolism, and enhance activity of dopamine, norepinephrine, and acetylcholine [36,144].

In a single-blind randomized study of 216 patients with severe or moderate TBI during the acute post-injury period, Calatayud et al. (1991) [23] observed improvements in motor, cognitive, and psychiatric function during treatment with CDP-choline, and use of this agent was associated with decreased length of hospitalization. Levin (1991) [90] performed a double-blind, placebo-controlled study of 14 patients to evaluate the efficacy of CDP-choline for treating postconcussional symptoms in the first month after mild to moderate TBI. Oral CDP-choline (1 g daily) reduced the severity of postconcussional symptoms and improved recognition memory for designs. Other aspects of neuropsychological performance were not significantly influenced by this treatment.

CDP-choline is available in the United States only as an over-the-counter nutritional supplement and is formulated most commonly in 250 mg capsules. A metanalysis of studies using CDP-choline in elderly patients suggests that its use is associated with fewer adverse effects than placebo [57], and there are no reports of serious adverse events related to treatment with this agent among persons with TBI. However, the lack of rigorous FDA scrutiny of the safety, tolerability, and efficacy of this "over-the-counter" agent require us to recommend caution and heightened clinical vigilance for adverse effects when CDP-choline is used in this or any clinical population.

## 9. Summary

Pharmacotherapy is one of several interventions for posttraumatic cognitive impairments and is at present, best regarded as an adjunct to nonpharmacologic therapies for such problems. Our present understanding of the neurochemistry of cognition and the neurochemical bases of posttraumatic cognitive impairments suggest that augmentation of cerebral catecholaminergic and cholinergic function are likely to be the most useful neurochemical targets for pharmacologic intervention in this population.

In general, persons with posttraumaic impairments in arousal, speed of processing, and attention may benefit most from treatment with a psychostimulant. Methylphenidate should be regarded as the first-line agent for this pupose. If methylphenidate proves ineffective or produces intolerable side effects, dextroamphetamine, amantadine, or bromocriptine may be useful alternative stimulant medications. In cases where none of these are effective, clinicians might consider use of modafanil, carbidopa/L-dopa, or other nonstandard agents with stimulating properties such as modafinil, protriptytline, or lamotrigine. Use of psychostimulants in the acute rehabilitation setting may facilitate engagement in rehabilitation therapies and it is possible that such treatment may hasten the recovery process (functionally, if not also neurobiologically). If used during the postacute injury period (during which spontaneous recovery may occur), periodically decreasing the dose of these agents after maximal cognitive benefit has been achieved is recommended in order to determine if continued prescription of a psychostimulant is still necessary. When used in the late post-injury period, common clinical experience suggests that these medications maintain their effectiveness over the long-term and that abuse of and/or dependency on these agents is rare.

Among persons whose predominant posttraumatic impairment is in the domain of memory (encoding, retrieval, or both), cholinesterase inhibitors may be of greatest benefit. Cholinergic augmentation would be predicted to have additional benefits on posttraumatic impairments in arousal, attention, language, executive function, and frontally-mediated behaviors, but the evidence supporting this suggestion is at present preliminary. Among the cholinesterase inhibitors, donepezil is used most commonly and is presently the agent with the most published evidence with which to support and guide its use. As with the psychostimulants, the cholinesterase inhibitors may improve both memory and the rate of functional improvement. If used during the period in which spontaneous recovery is likely to occur, periodic dose reduction and/or discontinuation of these agents is prudent in order to determine whether their use is still needed. In our experience, cognitive impairments that emerge following such dose reductions and/or medication discontinuations remain responsive to treatment once it is reinstituted.

In the absence of cost-effective and widely available *in vivo* markers of neurotransmitter function with which to guide the selection of a class of medication, treatment of posttraumatic cognitive impairments remains a matter of clinical judgment and empiric trial. Some persons with such impairments respond robustly to catecholaminergic agents, others to cholinesterase inhibitors, some require treatment with some combination of these agents, and others respond poorly to all presently available medications. Additional studies are needed to clarify which agents are most effective for which type of posttraumatic cognitive impairments and to facilitate the clinical identification of persons most likely to respond to each of these types of pharmacotherapy.

## Acknowledgments

This work was supported in part by HealthONE Spalding Rehabilitation Hospital.
